# Male osteoporosis: pathophysiology, risk assessment, and evidence-based management

**DOI:** 10.55730/1300-0144.6337

**Published:** 2026-05-20

**Authors:** Selin TEKİN, Alper GÜRLEK

**Affiliations:** Department of Endocrinology and Metabolism, Faculty of Medicine, Hacettepe University, Ankara, Turkiye

**Keywords:** Male osteoporosis, fracture in men, osteoporosis treatment in men

## Abstract

Osteoporosis in men is a common yet underrecognized condition associated with substantial fracture-related morbidity and excess mortality. Although men generally attain higher peak bone mass than women, age-related changes in bone microarchitecture, progressive cortical bone loss, declining sex steroid bioavailability, and a high prevalence of secondary causes contribute to a significant lifetime fracture risk. Importantly, a large proportion of fragility fractures in men occur at bone mineral density (BMD) values above the osteoporotic threshold, highlighting the limitations of BMD-based diagnosis alone. This review provides a comprehensive overview of the epidemiology, pathophysiology, and clinical characteristics of osteoporosis in men, with particular emphasis on sex-specific skeletal aging, hormonal regulation, and secondary osteoporosis. Current strategies for fracture risk assessment are critically examined, including the roles and limitations of dual-energy X-ray absorptiometry, vertebral imaging, the Fracture Risk Assessment Tool, and alternative risk prediction tools. We further summarize evidence-based approaches to management, encompassing lifestyle measures, correction of reversible risk factors, and pharmacological therapies. Antiresorptive agents remain the first-line treatment for most men, while anabolic therapies and sequential treatment strategies are increasingly recognized for those at very high risk of fracture. Overall, effective care in cases of male osteoporosis requires an integrated risk-based approach that extends beyond BMD to include clinical risk factors, secondary causes, and functional status. Improved recognition and sex-specific management strategies are essential to reduce the fracture burden and improve long-term outcomes in men.

## 1. Introduction

Osteoporosis has traditionally been regarded as a disease predominantly affecting women, a misconception that has contributed to the chronic underrecognition and undertreatment of osteoporosis in men [1]. Although men experience fewer fragility fractures than women overall, the absolute burden is substantial and clinically important. Approximately one in four men over the age of 45 years will sustain a major osteoporotic fracture during his lifetime, and men account for nearly 30% of all fragility fractures worldwide [1–5]. These data clearly indicate that osteoporosis in men is neither rare nor trivial.

Beyond fracture incidence, outcomes after osteoporotic fractures are disproportionately severe in men. Fracture-related morbidity and mortality particularly following hip fracture are consistently higher in men than in women, a disparity attributed to a higher burden of comorbidities, delayed diagnosis, underuse of osteoporosis therapies, and increased susceptibility to postoperative complications and infections [6–8]. Excess mortality may persist for more than a decade after the index fracture, underscoring the long-term systemic consequences of skeletal fragility in men [8].

Despite this substantial burden, osteoporosis care in men remains profoundly suboptimal. Screening, diagnosis, and treatment rates are consistently lower than in women, even after major fragility fractures that should prompt secondary prevention. Following vertebral or hip fractures, diagnostic rates in men are strikingly low compared to women, highlighting persistent systemic failures in the recognition and management of male osteoporosis [9,10].

Male osteoporosis is further distinguished by sex-specific patterns of skeletal aging. In men, age-related bone loss is characterized by trabecular thinning with relative preservation of connectivity, alongside progressive cortical thinning, increased intracortical porosity, and compensatory periosteal apposition changes that collectively reduce bone strength and increase fracture risk [11–14]. Nevertheless, osteoporosis management has historically focused on women, and guidance for men remains limited and fragmented. Although several male-specific or male-inclusive guidelines exist, intervention thresholds and treatment algorithms vary widely, reflecting the absence of a unified, evidence-based global consensus [9,15,16].

In this context, male osteoporosis constitutes a high-impact yet neglected public health problem. This review synthesizes current evidence on the epidemiology, pathophysiology, risk assessment, diagnosis, monitoring, and treatment of osteoporosis in men, integrating major guideline recommendations and recent advances in bone and endocrine research with the aim of providing a clinically pragmatic framework to improve recognition, management, and outcomes in this vulnerable population.

## 2. Literature search strategy

This is a narrative review. A comprehensive literature search was conducted using electronic databases without restrictions on publication date or language. Search terms included combinations of the following: “male osteoporosis,” “osteoporosis in men,” “bone mineral density,” “fragility fracture,” “fracture risk assessment,” “secondary osteoporosis,” “hypogonadism,” “androgen deprivation therapy,” “bisphosphonate,” “denosumab,” “teriparatide,” “romosozumab,” “Fracture Risk Assessment Tool” or “FRAX,” and “dual-energy X-ray absorptiometry” or “DXA.” Additional sources were identified through manual screening of the reference lists of the retrieved articles and relevant clinical practice guidelines, including those from the European Society for Clinical and Economic Aspects of Osteoporosis, Osteoarthritis and Musculoskeletal Diseases (ESCEO), the Endocrine Society, the International Osteoporosis Foundation (IOF), and the National Osteoporosis Guideline Group (NOGG). Priority was given to randomized controlled trials, prospective cohort studies, systematic reviews, metaanalyses, and major international guidelines. Studies specifically addressing male populations or reporting sex-disaggregated data were preferentially included.

## 3. Pathophysiology and sexual dimorphism in male osteoporosis

The substantial fracture burden and excess mortality observed in men with osteoporosis cannot be explained by bone mineral density (BMD) alone. Rather, male osteoporosis reflects distinct biological, hormonal, and structural mechanisms that differ fundamentally from those in women [13]. These sex-specific features influence skeletal development, patterns of age-related bone loss, fracture risk assessment, and therapeutic response, underscoring the need for a tailored approach to osteoporosis in men.

### 3.1. Skeletal development and peak bone mass

Men generally achieve higher peak bone mass and more favorable bone geometry than women, largely due to greater periosteal expansion during growth and puberty [17]. This process results in larger bone size, increased cortical thickness, and enhanced mechanical strength in early adulthood [18]. Testosterone plays a central role in stimulating periosteal apposition, while growth hormone and insulin-like growth factor-1 contribute to longitudinal bone growth [19]. Consequently, men enter adulthood with a structural advantage that may delay the development of osteoporosis later in life but does not prevent it, particularly in the presence of aging, hormonal decline, or secondary causes of bone loss [20].

### 3.2. Age-related bone loss: trabecular and cortical compartments

Both men and women experience a progressive decline in BMD with aging [21,22]. However, sexual dimorphism in skeletal aging provides a fundamental distinction between male and female osteoporosis [23]. In men, age-related trabecular bone loss occurs predominantly through trabecular thinning, with relative preservation of trabecular number and connectivity [13,24]. In contrast, women exhibit more pronounced disruption of trabecular connectivity, resulting in greater deterioration of the trabecular microarchitecture [24,25].

Cortical bone deterioration plays a particularly important role in skeletal fragility among men. Aging is accompanied by progressive cortical thinning, increased intracortical porosity, and expansion of the medullary cavity [26]. Although compensatory periosteal apposition may partially offset these changes, it is often insufficient to maintain overall bone strength. Importantly, cortical bone loss is strongly associated with hip and nonvertebral fractures, the fracture types that confer the greatest morbidity and mortality in men. Consequently, reliance on areal BMD measurements may underestimate fracture risk in men, particularly in clinical contexts where cortical bone deterioration predominates [12].

### 3.3. Lifelong effects of sex steroids on male bone

In later life, bone remodeling in men remains under hormonal control but follows a distinctly different trajectory than in women. Rather than experiencing an abrupt menopausal transition, men undergo a slow, progressive decline in bone mass with aging [33]. This process is accompanied by rising levels of sex hormone binding globulin (SHBG), resulting in reduced bioavailability of circulating sex steroids [33,34]. While associations between total testosterone levels and bone density have been inconsistent, accumulating evidence indicates that estradiol, particularly bioavailable estradiol, is more strongly linked to bone density, skeletal microarchitecture, and fracture risk in men [23,35]. Elevated SHBG has also emerged as an independent predictor of fracture risk. Collectively, these observations underscore that osteoporosis in men cannot be attributed solely to androgen deficiency; it reflects complex, lifelong interactions between androgenic and estrogenic signaling pathways influencing skeletal integrity [13].

### 3.4. The muscle–bone–falls axis

Skeletal fragility in men is closely linked to age-related declines in muscle mass, strength, and physical performance. Sarcopenia, frailty, and impaired balance substantially increase fall risk, a key determinant of fracture occurrence [36]. Hypogonadism, chronic inflammation, and comorbid conditions further exacerbate muscle loss, compounding fracture risk independently of BMD [37]. This muscle–bone–falls interaction provides a mechanistic explanation for the disproportionately severe postfracture complications and excess mortality observed in men [38,39].

## 4. Pathophysiological basis of secondary osteoporosis in men

**Figure 1:**
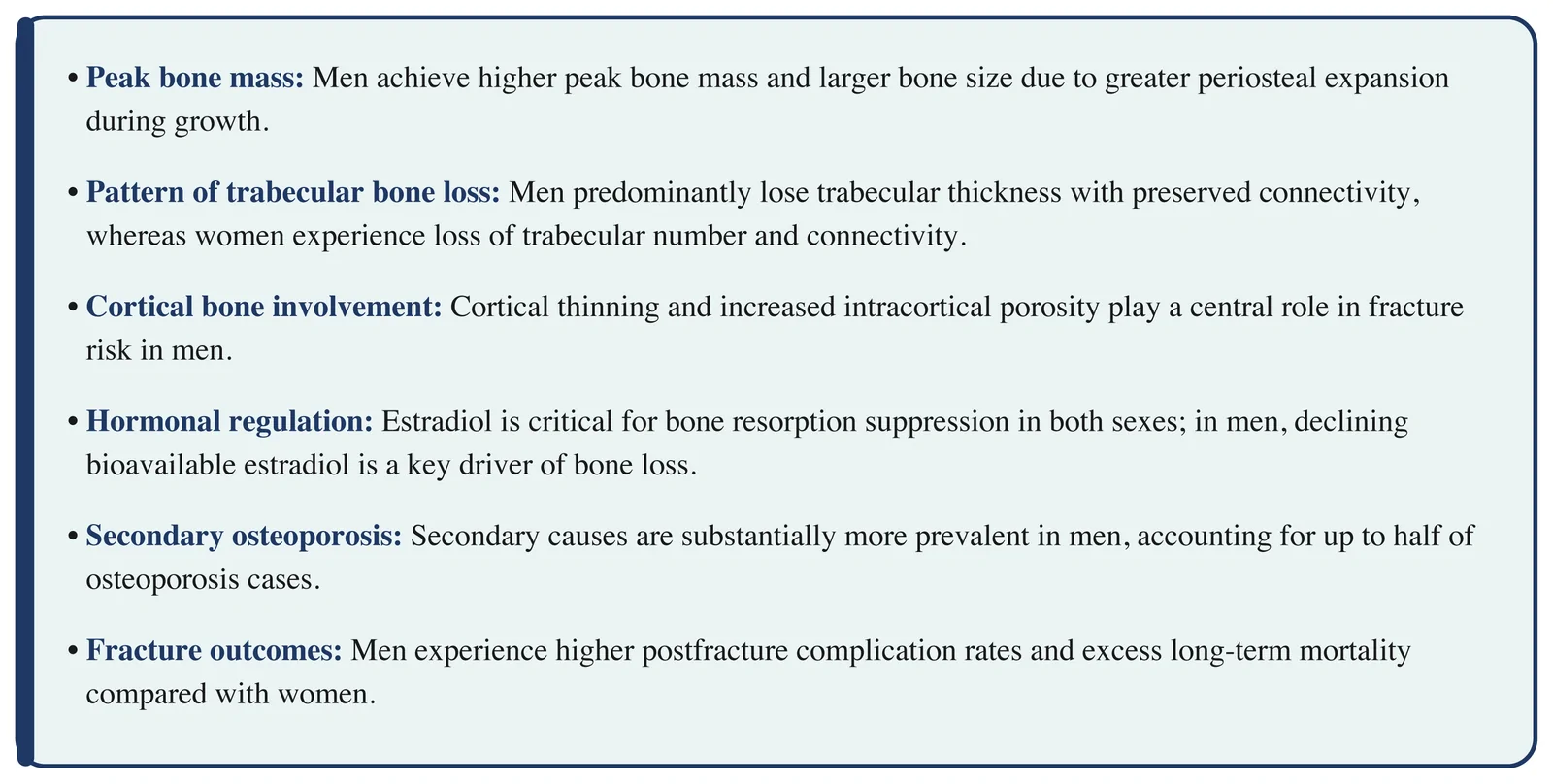
Key biological differences between male and female osteoporosis.

Secondary osteoporosis is substantially more prevalent in men than in women, and epidemiological data indicate that identifiable secondary causes are detectable in approximately 60% of men with osteoporotic fractures [40,41]. Hypogonadism, glucocorticoid exposure, excessive alcohol consumption, inflammatory diseases, and androgen deprivation therapy for prostate cancer exert profound effects on both trabecular and cortical bone [42]. Systematic evaluation for secondary causes is therefore a cornerstone of osteoporosis assessment in men (Figure 1).

### 4.1. Secondary osteoporosis in men: causes, evaluation, and clinical clues

The most frequent causes of secondary osteoporosis in men include [42–44]:

- **Hypogonadism**, both classical and functional, leading to reduced estradiol and testosterone bioavailability.
- **Glucocorticoid excess**, endogenous or exogenous, resulting in suppressed bone formation and increased resorption.
- **Chronic inflammatory diseases**, such as rheumatoid arthritis and inflammatory bowel disease.
- **Excessive alcohol consumption and smoking**, which impair osteoblast function and calcium homeostasis.
- **Endocrine disorders**, including hyperthyroidism, hyperparathyroidism, and Cushing syndrome.
- **Chronic kidney disease and malabsorption syndromes**, affecting mineral metabolism.
- **Androgen deprivation therapy for prostate cancer**, associated with rapid bone loss and high fracture risk.

Notably, multiple secondary factors frequently coexist, further compounding skeletal fragility.

### 4.2. Clinical clues suggesting secondary osteoporosis

Certain clinical features should prompt heightened suspicion for secondary osteoporosis in men. These include osteoporosis or fragility fractures occurring at a relatively young age, fractures in the absence of significant trauma, discordance between fracture risk and BMD values, rapid bone loss on serial dual-energy X-ray absorptiometry (DXA) measurements, and the presence of systemic symptoms such as weight loss, fatigue, or signs of hypogonadism. Vertebral fractures, which are often asymptomatic, are particularly important markers of underlying secondary disease [16].

## 5. Diagnostic evaluation: a systematic approach

**Figure 2:**
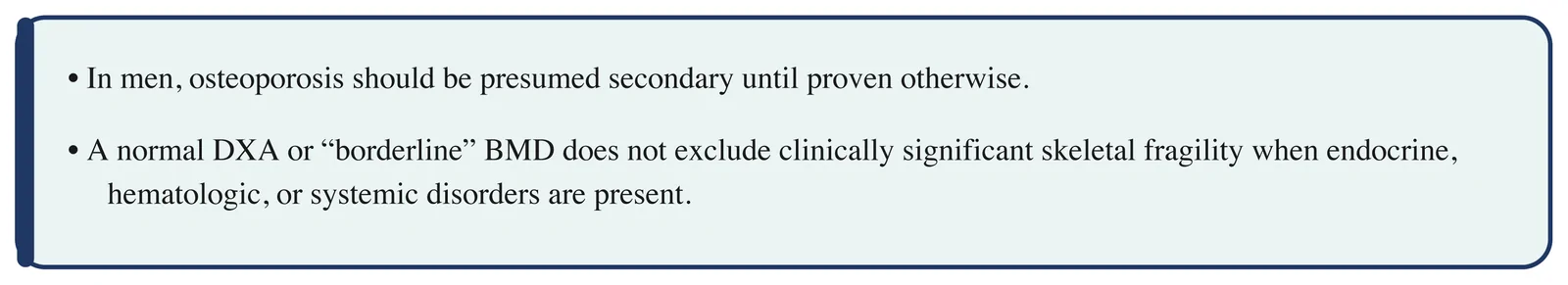
Endocrinologist’s approach to screening for secondary osteoporosis in men.

All men with osteoporosis or fragility fractures should undergo a structured laboratory evaluation aimed at identifying reversible contributors to bone loss. A minimal initial work-up should include serum calcium, phosphate, creatinine with estimated glomerular filtration rate, alkaline phosphatase, liver function tests, 25-hydroxyvitamin D, complete blood count, and total testosterone. Measurement of SHBG may provide additional insight into bioavailable sex steroid status (Figure 2) [16].

Further testing should be guided by clinical suspicion and may include parathyroid hormone, thyroid-stimulating hormone, cortisol assessment, celiac disease serology, and serum or urine protein electrophoresis. Importantly, the goal of evaluation is not merely diagnostic classification but identification of modifiable factors that can meaningfully alter fracture risk and therapeutic response (Table 1).

## 6. Screening and fracture risk assessment in men

Effective fracture prevention in men depends on accurate identification of those at increased risk. However, screening and risk assessment strategies for osteoporosis in men remain less clearly defined than in women, reflecting limited sex-specific evidence and heterogeneity across guidelines. This uncertainty has contributed to delayed diagnosis and treatment, particularly in men without classical risk factors.

### 6.1.Who should be screened?

Most guidelines recommend a risk-based rather than population-wide screening approach in men. BMD testing is generally advised for men aged ≥70 years and for younger men (≥50 years) with clinical risk factors such as prior fragility fracture, hypogonadism, chronic glucocorticoid use, inflammatory disease, excessive alcohol intake, or conditions associated with secondary osteoporosis [45].

Importantly, men presenting with a fragility fracture should be considered at high fracture risk irrespective of BMD and should undergo comprehensive evaluation without delay. From an endocrine perspective, screening decisions should prioritize clinical context over chronological age alone. Men with endocrine disorders, rapid bone loss, or fractures disproportionate to BMD values warrant early assessment even if they fall below conventional age thresholds.

### 6.2. Role and limitations of DXA

DXA remains the cornerstone of osteoporosis diagnosis and a key component of fracture risk assessment in men. The World Health Organization (WHO) definition of osteoporosis, based on a T-score threshold of −2.5 derived from the female National Health and Nutrition Examination Survey reference population, has long raised questions regarding its applicability to men [46]. Nevertheless, evidence indicates that for a given absolute BMD, hip fracture risk and the gradient of fracture risk with declining BMD are broadly similar in men and women, supporting the use of a unified densitometric threshold [46,47]. However, the contribution of BMD to fracture risk diminishes with age, and substantial geographic variation in fracture incidence exists despite relatively modest differences in population BMD [48,49]. Moreover, low BMD performs poorly as a population screening tool, as most fragility fractures occur in individuals with BMD values above the osteoporotic threshold.

DXA assessment of the lumbar spine and hip is recommended in all men evaluated for osteoporosis. Forearm DXA of the nondominant arm should be considered when spine or hip measurements are unreliable due to degenerative changes or artifacts, and they should be routinely performed, when feasible, in patients with hyperparathyroidism. Vertebral fracture assessment (VFA) is strongly encouraged given the high prevalence of asymptomatic vertebral fractures in men [48,49].

Despite its central role, DXA has important limitations in male osteoporosis. Age-related bone loss in men predominantly affects cortical bone through increased intracortical porosity, which is not adequately captured by areal BMD measurements. Consequently, fragility fractures may occur at BMD levels above conventional thresholds, and degenerative spinal changes or vascular calcifications may artifactually elevate spine BMD. These limitations underscore the need to interpret DXA findings alongside clinical risk factors, fracture history, and adjunctive imaging to achieve accurate fracture risk stratification in men [16].

### 6.3. The FRAX in men: strengths, shortcomings, and alternative risk assessment tools

The Fracture Risk Assessment Tool (FRAX) is widely used to estimate 10-year probabilities of major osteoporotic and hip fractures in men and women by integrating clinical risk factors with or without femoral neck BMD, and optionally the trabecular bone score (TBS) [50,51]. Extending fracture risk assessment beyond BMD alone, the FRAX is endorsed by multiple international guidelines for informing treatment decisions in men [16,52,53]. The clinical value of the FRAX depends on clearly defined intervention thresholds linking fracture probability to treatment. Fixed densitometric thresholds, such as T-score ≤ −2.5, become less informative with advancing age, whereas a single fixed fracture-probability threshold risks undertreating younger individuals and overtreating older ones. To address these limitations, the ESCEO, IOF, and NOGG recommend age-dependent, country-specific FRAX thresholds anchored to the fracture probability associated with a prior fragility fracture, thereby accounting for age-related changes and competing mortality risk [45,54]. On the basis of equity and comparable cost-effectiveness, identical intervention thresholds are recommended for men and women [45].

Despite these strengths, the FRAX has notable limitations in male osteoporosis. It does not incorporate fall risk, sarcopenia, dose-dependent glucocorticoid exposure, or the severity of secondary osteoporosis and may therefore underestimate fracture risk in men with rapid bone loss, multiple vertebral fractures, or androgen deprivation therapy. Consequently, FRAX probabilities should be interpreted as risk estimates rather than absolute treatment cut-offs, and clinical judgment remains essential. Proposed refinements include adjustment for glucocorticoid dose, consideration of spine–hip BMD discordance, and incorporation of the TBS.

The TBS, derived from lumbar spine DXA texture analysis, provides an indirect assessment of bone microarchitecture independent of BMD and has been validated as an adjunct to the FRAX in men. A lower TBS is associated with increased fracture risk even at nonosteoporotic BMD values, and TBS-adjusted FRAX probabilities may more accurately identify high-risk individuals. Other emerging tools, including high-resolution peripheral quantitative computed tomography and bone microarchitecture indices, support the detailed evaluation of cortical and trabecular compartments but remain research instruments not yet in routine clinical use. Alternative tools such as the Garvan Fracture Risk Calculator and QFracture, which incorporate fall history, may better capture short-term fracture risk in selected men, while the Male Osteoporosis Risk Estimation Score may assist in identifying candidates for DXA testing in primary care [55–58]. Overall, because fracture risk at a given BMD is similar in men and women and most fractures occur above the osteoporotic BMD range, current guidelines advocate an integrated risk-based approach combining DXA, FRAX, and complementary tools, with particular attention to secondary osteoporosis and fall risk [16] (Table 2).

### 6.4. Vertebral fractures: a missed opportunity

Vertebral fractures are common, often asymptomatic, and strong predictors of future fractures and mortality in men, yet they are frequently underdiagnosed [4]. Identification of vertebral fractures by DXA-based VFA or lateral spine radiography can substantially reclassify fracture risk and alter management, even in men with nonosteoporotic BMD [48,49]. Detection of a vertebral fracture should place a man in a high-risk category and prompt pharmacological treatment regardless of BMD; VFA is preferred when available, with lateral spine radiographs as an alternative.

### 6.5. Comparison of major guidelines for male osteoporosis

Major international guidelines provide frameworks for osteoporosis management in men, with notable areas of concordance and divergence (Table 3). The ESCEO/IOF guideline [16] recommends BMD testing in men aged ≥70 years and younger men with risk factors, endorses age-dependent FRAX intervention thresholds anchored to the fracture probability of a prior fragility fracture, and advocates identical treatment thresholds for men and women on grounds of equity. The Endocrine Society guideline [52] similarly recommends pharmacological treatment for men with T-score ≤ −2.5, prior fragility fracture, or high FRAX probability, and this guideline specifically emphasizes evaluation and correction of underlying hypogonadism and other secondary causes as integral to management. The NOGG 2024 guideline [54] applies country-specific, age-adjusted FRAX thresholds derived from UK fracture epidemiology, recommending treatment when the 10-year probability of major osteoporotic fracture equals or exceeds that associated with a prior fragility fracture at the same age, and the 2024 update strengthens recommendations on anabolic-first therapy for very high risk. Across all three guidelines, bisphosphonates are endorsed as first-line therapy and anabolic agents are reserved for very high fracture risk. Key divergences are related to intervention thresholds, the role of testosterone replacement, the integration of the FRAX and clinical risk factors, and the level of male-specific evidence cited. Clinicians should select the guideline most applicable to their regional fracture epidemiology and clinical context.

### 6.6. Toward an endocrine-informed risk assessment strategy

**Figure 3:**
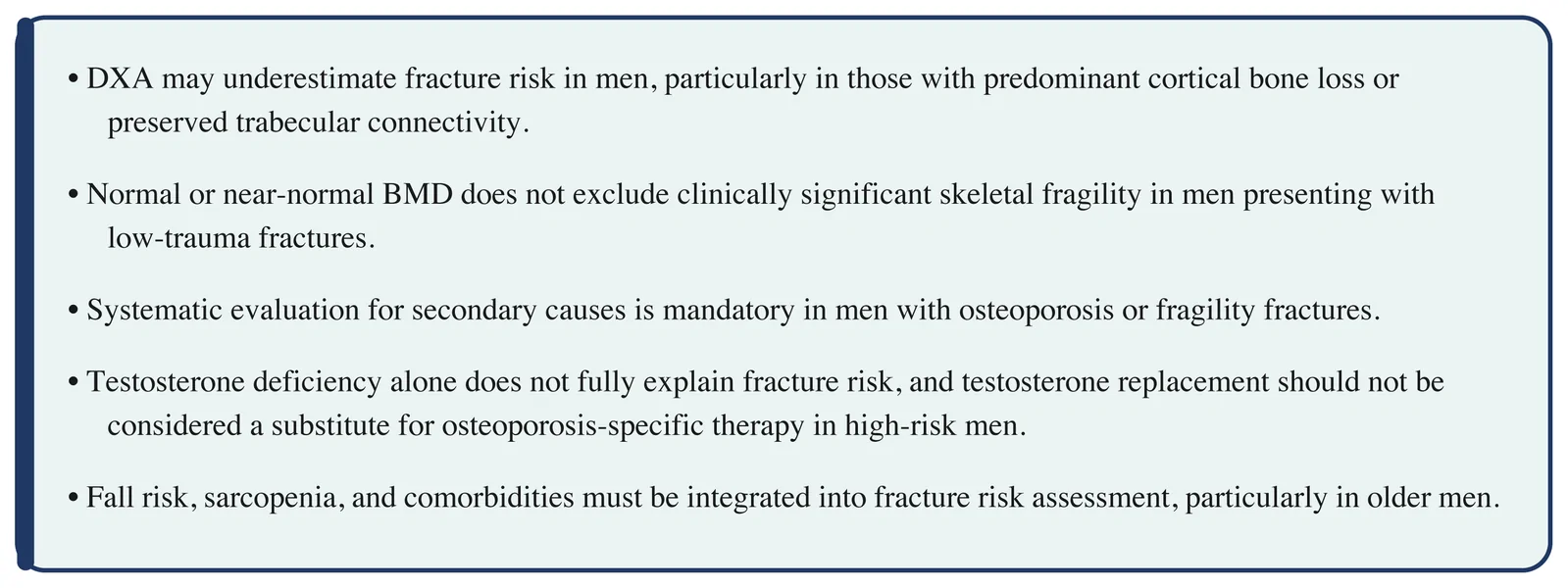
Fracture risk assessment in men.

Optimal fracture risk assessment in men should integrate BMD with clinical risk factors, secondary causes of osteoporosis, and functional status. A holistic approach is essential, incorporating hormonal status, comorbidities, fall risk, and indicators of bone quality beyond BMD. Men with fractures disproportionate to BMD, rapid bone loss, or endocrine disorders require individualized evaluation and should not be reassured by nonosteoporotic DXA values (Figure 3).

In clinical practice, the main challenge is not the diagnostic definition of osteoporosis but the failure to identify and treat men at high fracture risk. The WHO BMD-based definition provides a robust and widely accepted framework for diagnosis and research; however, diagnostic thresholds should not be confused with treatment thresholds. A fragility fracture is a clinical event that warrants secondary prevention regardless of BMD. Requiring DXA confirmation before treatment may delay care and contribute to treatment gaps, particularly in men. Therefore, risk-based strategies that combine clinical factors such as prior fracture, age, comorbidities, and fall risk with BMD offer a more practical and effective approach to fracture prevention in men [48] (Table 2).

## 7. Treatment of osteoporosis in men

A summary of treatment recommendations according to fracture risk category is presented in Table 4. Current recommendations support a comprehensive approach to the management of osteoporosis in both men and women. Men with osteoporosis (based on BMD T-score ≤ −2.5 or BMD T-score between −1.0 and −2.5 and the presence of a fragility fracture) and men with osteopenia and a FRAX 10-year probability threshold of ≥3% for hip fracture and ≥20% for major osteoporotic fracture should receive osteoporosis treatment [16,45,52].

The primary goal of osteoporosis treatment in men is the prevention of fragility fractures, particularly hip and vertebral fractures, which are associated with substantial morbidity and excess mortality. Although evidence in men is less extensive than in women, available randomized trials, observational studies, and guideline recommendations support the use of antiresorptive and anabolic therapies in men at high fracture risk [48]. Importantly, treatment decisions should be guided by absolute fracture risk, the presence of prior fragility fractures, and underlying secondary causes rather than BMD alone [16].

## 8. General principles of treatment

Men with a prior fragility hip or vertebral fracture should be considered to be at very high risk of fracture and should be treated without delay regardless of BMD [5,9,16]. In men without prior fractures, pharmacological therapy is indicated when fracture risk assessed by the FRAX or equivalent tools is high, or when BMD is in the osteoporotic range in the presence of additional risk factors [16,45,52,53]. Correction of reversible contributors, including vitamin D deficiency, inadequate calcium intake, hypogonadism (when clinically indicated), and excessive glucocorticoid exposure, should accompany but not replace osteoporosis-specific pharmacological therapy in high-risk men.

**Figure 4:**
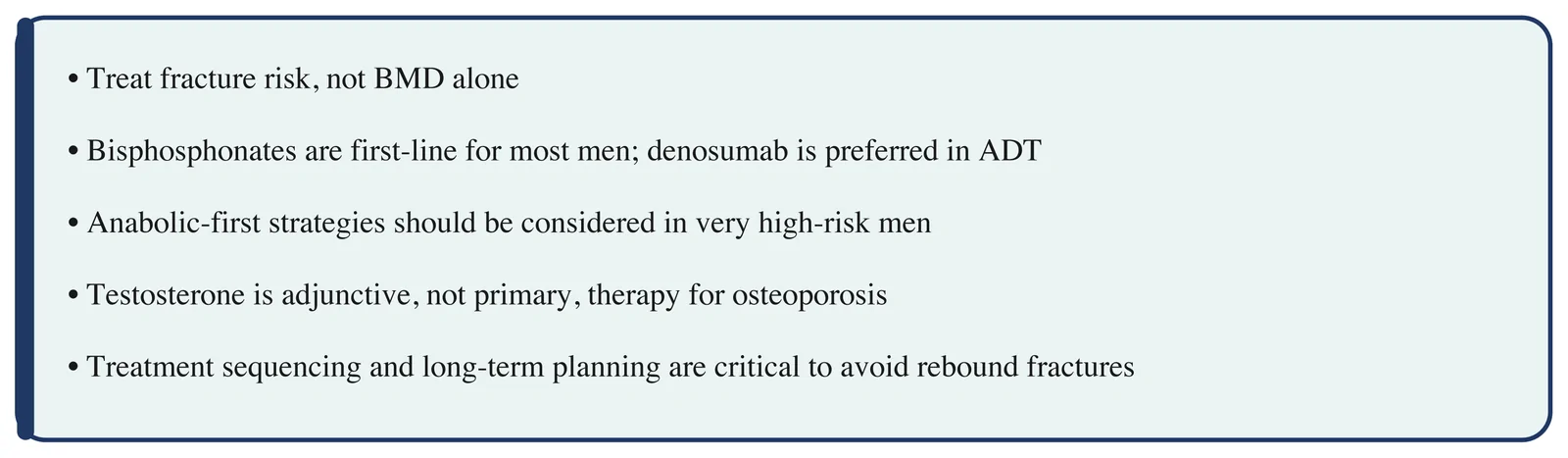
Clinical approach to the treatment of osteoporosis in men — Who should be treated immediately?

Adequate calcium and vitamin D intake is a fundamental component of osteoporosis management in men. A daily intake of at least 800 IU of vitamin D and 1000–1200 mg of calcium from dietary and/or supplemental sources is recommended to support bone mineralization and fracture prevention while avoiding excessive supplementation [59,60]. In addition, lifestyle measures including smoking cessation, moderation of alcohol intake, regular weight-bearing and resistance exercise, and adequate dietary protein play an important adjunctive role by improving bone and muscle health and reducing fall and fracture risk [61,62]. These nonpharmacological interventions should be routinely implemented alongside pharmacological therapy to optimize skeletal outcomes in men with osteoporosis (Figure 4).

### 8.1. Antiresorptive therapies

Bisphosphonates constitute the cornerstone of antiresorptive therapy in men with osteoporosis and remain the preferred first-line pharmacological option owing to their established efficacy, safety, and cost-effectiveness. Among oral agents, alendronate and risedronate have the most robust evidence, demonstrating consistent increases in lumbar spine (LS), femoral neck (FN) [63], and total hip (TH) BMD, together with significant reductions in vertebral fracture risk [15].

In randomized controlled trials, alendronate increased LS BMD by approximately 5%–7% and FN BMD by 2%–5% over 2 years, with sustained gains and up to a 57% reduction in vertebral fracture risk in longer-term studies [64–68]. Risedronate showed comparable efficacy, with significant BMD improvements at major skeletal sites and reductions of ~60% in vertebral and ~45% in nonvertebral fractures over 2 years [69–71]. Ibandronate has demonstrated more modest but significant BMD gains in the spine and hip [72,73].

Intravenous zoledronic acid (5 mg annually) provides an effective alternative to oral bisphosphonates, particularly in men with gastrointestinal intolerance, adherence issues, or recent hip fracture. Zoledronic acid increased LS BMD by ~6% and hip BMD by 2%–3% over 24 months and reduced morphometric vertebral fracture risk by up to 67% [74], although effects on clinical fracture outcomes after hip fracture repair were less consistent [75].

Denosumab, a monoclonal antibody against receptor activator of nuclear factor kappa-B ligand (RANKL), is an effective nonbisphosphonate antiresorptive agent. In men with low BMD or osteoporosis, denosumab (60 mg every 6 months) significantly increased BMD at the spine, hip, and distal radius [76]. It is particularly useful in men with bisphosphonate intolerance, in selected cases of chronic kidney disease, and in those receiving androgen deprivation therapy for prostate cancer, where vertebral fracture risk reduction has been demonstrated. However, treatment discontinuation is associated with rapid bone loss, underscoring the need for appropriate transition strategies; therefore, sequential therapy with a bisphosphonate is mandatory if denosumab is stopped.

Overall, strong evidence supports the use of bisphosphonates and denosumab as the foundation of pharmacological management of osteoporosis in men, with agent selection guided by fracture risk, comorbidities, tolerability, and patient preference. Clinical considerations include the following: renal function must be assessed prior to initiation, long-term therapy requires periodic reassessment of fracture risk, and drug holidays may be considered in men at low-to-moderate risk after prolonged use.

### 8.2. Anabolic therapies and treatment sequencing

In men with imminent or very high fracture risk, anabolic agents are recommended as first-line therapy and should be followed by antiresorptive treatment to consolidate and maintain skeletal gains. Although no universal definition exists, very high fracture risk is commonly considered in men with markedly elevated fracture probabilities (e.g., a 10-year major osteoporotic fracture risk of >30% or hip fracture risk of >4%–5%), recent or multiple fragility fractures, or severe osteoporosis accompanied by additional clinical risk factors [53].

Anabolic agents including teriparatide, abaloparatide, and romosozumab primarily stimulate bone formation, leading to rapid and substantial increases in BMD and clinically meaningful reductions in fracture risk. Growing evidence supports a sequential treatment strategy in which an initial course of anabolic therapy is followed by antiresorptive treatment, such as a bisphosphonate or denosumab, to preserve and consolidate BMD gains. This approach appears particularly effective in men with severe osteoporosis or advanced skeletal fragility.

The efficacy of teriparatide in men has been demonstrated in randomized controlled trials. In a double-blind study with a median treatment duration of 11 months, teriparatide significantly increased BMD at the lumbar spine, femoral neck, and total hip compared with placebo [77]. Follow-up analyses showed sustained BMD benefits at the lumbar spine and hip up to 30–42 months after treatment initiation [78]. Notably, pooled teriparatide-treated groups experienced an 83% reduction in new moderate or severe vertebral fractures compared with placebo [79]. Smaller studies using human parathyroid hormone [1–34] have reported even greater lumbar spine BMD gains, with modest improvements at cortical sites.

Abaloparatide has also shown efficacy in men with osteoporosis. In a randomized controlled trial, daily subcutaneous abaloparatide administered for 12 months resulted in significant BMD increases at the lumbar spine, femoral neck, and total hip compared to a placebo, with response magnitudes comparable to those observed in postmenopausal women [80,81]. Similarly, romosozumab has demonstrated rapid and pronounced skeletal effects in men. In a randomized, double-blind study, monthly romosozumab increased LS BMD by approximately 11% within 12 months, with significant gains at the TH and FN evident as early as 6 months [82]. These findings reflect its dual anabolic and antiresorptive mechanism of action.

Overall, anabolic therapies are the treatment of choice for men at very high fracture risk, particularly those with multiple vertebral fractures, very low BMD, or fractures occurring despite prior antiresorptive therapy. When followed by appropriate antiresorptive maintenance, this sequential strategy provides an effective framework for maximizing BMD gains and reducing fracture risk in men with severe osteoporosis [83].

## 9. Key Principles of Sequential Therapy

- Anabolic therapy → antiresorptive consolidation
- Avoid direct transition from denosumab to teriparatide
- Individualize treatment duration based on fracture risk, response, and tolerability

### 9.1. Testosterone replacement therapy: where does it fit?

Testosterone replacement therapy (TRT) improves BMD in hypogonadal men but has not been shown to reduce fracture risk [84,85]. Current evidence indicates that testosterone alone is insufficient to prevent fractures in men at high risk and should not be considered a primary osteoporosis treatment [85].

From an endocrine perspective, TRT may be considered in men with symptomatic, confirmed hypogonadism. TRT does not replace osteoporosis-specific therapy in men with fragility fractures or high fracture risk. In hypogonadal men at high fracture risk, TRT may be used in combination with antiresorptive or anabolic therapy. This distinction is critical, as reliance on testosterone alone may delay effective fracture prevention.

### 9.2. Osteoporosis in men receiving androgen deprivation therapy

Androgen deprivation therapy (ADT) is associated with rapid bone loss and a marked increase in fracture risk, often within the first year of treatment. Men initiating ADT should undergo baseline fracture risk assessment and early pharmacological intervention when indicated. Denosumab and bisphosphonates are both effective in this setting, with denosumab demonstrating robust vertebral fracture risk reduction. In a large randomized trial involving 1468 men with nonmetastatic hormone-sensitive prostate cancer receiving androgen deprivation therapy, denosumab (60 mg every 6 months) significantly increased bone mineral density and reduced vertebral fracture risk by 62% over 3 years, with fracture rates of 1.5% in the denosumab group compared to 3.9% in the placebo group [86]. A 2023 metaanalysis showed that denosumab significantly increased BMD in men, with mean improvements of 5.8% at the lumbar spine, 2.1% at the femoral neck, and 2.3% at the total hip, with all effects reaching statistical significance [87]. Early intervention is essential, as waiting for BMD to decline into the osteoporotic range may miss a critical window for fracture prevention.

## 10. Monitoring and long-term management

Treatment response should be monitored with repeat DXA every 1–2 years, with longer intervals once stability is achieved [52]. Bone turnover markers, such as procollagen type I N-terminal propeptide (P1NP) and C-terminal telopeptide of type I collagen (CTX), provide complementary information on bone formation and resorption and can be used to monitor response to osteoporosis therapy, although their interpretation is limited by biological variability and lack of standardized reference ranges [88,89]. Long-term management requires periodic reassessment of fracture risk, comorbidities, and treatment tolerability. As in women, osteoporosis in men should be approached as a chronic disease requiring sustained attention rather than a finite course of therapy.

## 11. Knowledge gaps, controversies, and future directions

Despite growing awareness, major gaps persist in the recognition, screening, and management of osteoporosis in men. Screening and treatment rates remain low, male-specific guidelines are limited, and men continue to be underrepresented in epidemiological studies and randomized trials, resulting in insufficient fracture outcome data particularly for nonvertebral fractures and uncertainty regarding optimal treatment strategies. Ongoing controversies include the lack of evidence supporting population-wide screening in men, the unclear impact of TRT on fracture risk despite its effects on BMD, and the limited male-specific data supporting anabolic-first treatment approaches. These gaps are compounded by suboptimal postfracture evaluation and secondary prevention, contributing to excess fracture-related morbidity and mortality in men. Future priorities include the development of male-specific screening and treatment guidelines; randomized trials powered for fracture outcomes; improved integration of fall risk, sarcopenia, and endocrine disorders into risk assessment models; and expanded evaluation of anabolic and combination therapies. Addressing these deficiencies is essential to shift male osteoporosis care from extrapolation toward evidence-based, sex-specific management.

## 12. Conclusion

Osteoporosis in men is common, underdiagnosed, and associated with substantial morbidity and excess mortality. Biological differences in skeletal aging, the high prevalence of secondary causes, and limitations of existing diagnostic and risk assessment tools contribute to delayed recognition and undertreatment. Fracture risk in men should be assessed using an integrated, endocrine-informed approach that extends beyond bone mineral density alone.

Effective management requires early identification of high-risk individuals, systematic evaluation for secondary causes, and timely initiation of appropriately sequenced pharmacological therapy. Bisphosphonates and denosumab remain the cornerstone of treatment for most men, but anabolic-first strategies should be considered in those at very high fracture risk. TRT has a limited adjunctive role and should not replace osteoporosis-specific treatment.

Closing the care gap in male osteoporosis will require dedicated research, improved risk stratification strategies, and the development of male-specific clinical guidelines. Recognizing osteoporosis in men as a distinct clinical entity rather than a variant of postmenopausal osteoporosis is essential to reducing fracture burden and improving long-term outcomes in this underserved population.

## Figures and Tables

**Table 1:** Secondary causes of and systemic approach to male osteoporosis.

Secondary cause	Clinical clues	Recommended tests	Endocrinology-oriented notes
Hypogonadism	Low libido, erectile dysfunction, sarcopenia, anemia, unexplained fractures	Morning total testosterone, SHBG, calculated free testosterone	Normal total testosterone may be misleading if SHBG is elevated
Glucocorticoid excess	Chronic steroid use, central obesity, diabetes, hypertension, vertebral fractures	Medication history; overnight dexamethasone suppression test (if Cushing syndrome is suspected)	Bone loss may occur early, even with moderate doses
Hyperthyroidism	Weight loss, palpitations, atrial fibrillation, cortical bone loss	TSH ± free T4	Subclinical hyperthyroidism increases fracture risk
Primary hyperparathyroidism	Hypercalcemia, nephrolithiasis, predominant cortical bone loss	Serum calcium, PTH, 25-hydroxyvitamin D	May present with normal DXA spine but low cortical BMD
Vitamin D deficiency / osteomalacia	Bone pain, muscle weakness, low-trauma fractures	25-hydroxyvitamin D, calcium, phosphate, ALP	Very common; often coexists with other secondary causes
Chronic kidney disease (CKD-MBD)	Reduced eGFR, vascular calcification, abnormal calcium–phosphate balance	Creatinine, eGFR, calcium, phosphate, PTH	Fracture risk increases even at early CKD stages
Chronic inflammatory diseases	Long-standing inflammation, steroid exposure	CRP, ESR, disease-specific evaluation	Inflammation and treatment synergistically increase bone loss
Alcohol excess	High alcohol intake, liver disease	Liver function tests, clinical history	Direct toxic effect on osteoblasts
Smoking	Long smoking history	Clinical history	Independent risk factor for fractures
Malabsorption / celiac disease	Chronic diarrhea, low BMI, iron deficiency anemia	tTG-IgA ± total IgA	To be considered even in the absence of GI symptoms
Monoclonal gammopathy / multiple myeloma	Back pain, anemia, renal dysfunction	Serum ± urine protein electrophoresis	Must be excluded in unexplained vertebral fractures
Systemic mastocytosis	Severe or early-onset osteoporosis, multiple vertebral fractures	Baseline serum tryptase	Often overlooked; elevated tryptase warrants hematological referral
Androgen deprivation therapy (ADT)	Prostate cancer, rapid bone loss	Treatment history, baseline DXA	Bone loss begins early after ADT initiation

SHBG: Sex hormone-binding globulin; PTH: parathyroid hormone; TSH: thyroid-stimulating hormone; T4: thyroxine; ALP: alkaline phosphatase; eGFR: estimated glomerular filtration rate; CKD-MBD: chronic kidney disease–mineral and bone disorder; CRP: C-reactive protein; ESR: erythrocyte sedimentation rate; BMI: body mass index; tTG-IgA: tissue transglutaminase immunoglobulin A; DXA: dual-energy X-ray absorptiometry; BMD: bone mineral density; GI: gastrointestinal; ADT: androgen deprivation therapy.

**Table 2:** Risk assessment tools in male osteoporosis.

Tool	Designed for men	Includes BMD	Includes falls	Primary clinical use in men	Key strengths	Key limitations
FRAX	Yes	Optional (femoral neck)	No	Estimation of 10-year probability of major osteoporotic and hip fractures; treatment decisions	Widely validated; integrates mortality risk; guideline-endorsed	Underestimates risk in men with falls, sarcopenia, severe secondary osteoporosis, ADT, or rapid bone loss
Garvan Fracture Risk Calculator	Yes	Yes	Yes	Short- to medium-term fracture risk including falls	Incorporates fall history; useful in frail older men	Less widely used; limited guideline integration
QFracture	Yes	No	Yes	Primary care fracture risk assessment	Includes falls, comorbidities, medications	qNo BMD input; population-specific (UK-based)
MORES	Male-specific	No	No	Prescreening tool to identify men who should undergo DXA	Simple; uses age, weight, COPD history	Not a fracture prediction tool; cannot guide treatment
DXA (BMD alone)	Yes	Yes	No	Diagnosis and monitoring of osteoporosis	Standardized, reproducible	Low sensitivity; most fractures occur above T-score of −2.5

FRAX: Fracture Risk Assessment Tool; BMD: bone mineral density; DXA: dual-energy X-ray absorptiometry; MORES: Male Osteoporosis Risk Estimation Score; ADT: androgen deprivation therapy; COPD: chronic obstructive pulmonary disease.

**Table 3:** Comparison of major international guidelines for male osteoporosis.

Guideline	Screening indication	Treatment threshold	Male-specific features
ESCEO/IOF [16]	Men aged ≥70 years, or younger with risk factors	Age-adjusted FRAX threshold = fracture probability of prior fragility fracture at same age; equal thresholds for men and women	First dedicated male guideline from ESCEO; endorses TBS-adjusted FRAX; emphasizes identical intervention equity
Endocrine Society [52]	Men aged ≥70 years, or aged 50–69 with risk factors	T-score ≤ −2.5; prior hip/spine fracture; or high FRAX probability	Explicit hypogonadism evaluation; testosterone replacement as adjunct (not primary therapy); ADT-related fracture risk addressed
NOGG 2024 [54]	Based on FRAX clinical risk factor assessment; DXA for intermediate risk	Country-specific, age-dependent FRAX threshold (UK data); assessment pathway = intervention threshold at prior fracture probability	Updated 2024: strengthened anabolic-first recommendation for very high risk; fall assessment integration

ESCEO: European Society for Clinical and Economic Aspects of Osteoporosis, Osteoarthritis and Musculoskeletal Diseases; IOF: International Osteoporosis Foundation; NOGG: National Osteoporosis Guideline Group; FRAX: Fracture Risk Assessment Tool; DXA: dual-energy X-ray absorptiometry; TBS: trabecular bone score; ADT: androgen deprivation therapy.

**Table 4:** Treatment recommendations according to fracture risk category in men with osteoporosis.

Risk category	Definition	Recommended first-line therapy	Notes
Low risk	T-score > −2.5, no prior fracture, low FRAX probability (10-year MOF < 10%, hip < 1%)	Nonpharmacological measures (calcium, vitamin D, exercise, fall prevention)	Reassess in 2–3 years
High risk	T-score ≤ −2.5 without prior fracture, or osteopenia + high FRAX (MOF ≥ 20%, hip ≥ 3%)	Bisphosphonate (alendronate or zoledronic acid); denosumab as alternative	Correct secondary causes; monitor with DXA every 1–2 years
Very high risk	Prior hip or vertebral fracture, or multiple fractures, or T-score ≤ −3.0 + high FRAX, or fracture during therapy	Anabolic agent first (teriparatide, abaloparatide, or romosozumab), followed by antiresorptive consolidation	Do not stop denosumab without bisphosphonate transition; avoid direct denosumab → teriparatide switch

MOF: Major osteoporotic fracture; DXA: dual-energy X-ray absorptiometry; FRAX: Fracture Risk Assessment Tool. Risk categories adapted from ESCEO/IOF and Endocrine Society guidelines.
